# Illegal commercial promotion of products competing with breastfeeding

**DOI:** 10.11606/s1518-8787.2020054000854

**Published:** 2020-01-23

**Authors:** Karine Borges da Silva, Maria Inês Couto de Oliveira, Cristiano Siqueira Boccolini, Enilce de Oliveira Fonseca Sally

**Affiliations:** I Universidade Federal Fluminense Programa de Pós-graduação em Saúde Coletiva NiteróiRJ Brasil Universidade Federal Fluminense. Programa de Pós-graduação em Saúde Coletiva. Niterói, RJ, Brasil; II Universidade Federal Fluminense Instituto de Saúde Coletiva Departamento de Epidemiologia e Bioestatística NiteróiRJ Brasil Universidade Federal Fluminense. Instituto de Saúde Coletiva. Departamento de Epidemiologia e Bioestatística. Niterói, RJ, Brasil; III Fundação Oswaldo Cruz Instituto de Comunicação e Informação Científica e Tecnológica em Saúde Laboratório de Informação em Saúde Rio de JaneiroRJ Brasil Fundação Oswaldo Cruz. Instituto de Comunicação e Informação Científica e Tecnológica em Saúde. Laboratório de Informação em Saúde. Rio de Janeiro, RJ, Brasil; IV Universidade Federal Fluminense Faculdade de Nutrição Departamento de Nutrição Social NiteróiRJ Brasil Universidade Federal Fluminense. Faculdade de Nutrição. Departamento de Nutrição Social. Niterói, RJ, Brasil

**Keywords:** Breast-Milk substitutes, Infant Formula, Pacifiers, Products Commerce, Direct-to-Consumer Advertising

## Abstract

**OBJECTIVE:**

To assess if the commercialization of infant formulas, baby bottles, bottle nipples, pacifiers and nipple protectors is performed in compliance with the *Norma Brasileira de Comercialização de Alimentos para Lactentes e Crianças de Primeira Infância e de Produtos de Puericultura Correlatos* (NBCAL – Brazilian Code of Marketing of Infant and Toddlers Food and Childcare-related products). The commercial promotion of these products is prohibited by the Law 11,265.

**METHOD:**

Cross-sectional study conducted in 2017 through a census of all pharmacies, supermarkets and department stores that sold products covered by NBCAL in the South Zone of Rio de Janeiro. Health professionals trained at NBCAL used structured electronic form for direct observation of establishments and for interviews with their managers. We created indicators to evaluate commercial practices and performed descriptive analyses.

**RESULTS:**

A total of 352 commercial establishments were evaluated: 240 pharmacies, 88 supermarkets and 24 department stores, of which 88% sold products whose promotion is prohibited by NBCAL. Illegal commercial promotions were found in 20.3% of the establishments that sold the products we investigated: 52 pharmacies (21.9%), four supermarkets (7.5%) and seven department stores (33.3%). The most frequent commercial promotion strategies were discounts (13.2%) and special exposures (9.3%). The products with the highest prevalence of infractions of NBCAL were infant formulas (16.0%). We interviewed 309 managers of commercial establishments; 50.8% reported unfamiliarity with the law. More than three-quarters of the managers reported having been visited at the establishments by commercial representatives of companies that produce infant formulas.

**CONCLUSION:**

More than a fifth of commercial establishments promoted infant formulas, baby bottles and nipples, although this practice has been banned in Brazil for thirty years. We think it is necessary to train those managers. Government agencies must monitor commercial establishments in order to inhibit strategies of persuasion and induction to sales of these products, ensuring mothers’ autonomy in the decision on the feeding of their children.

## INTRODUCTION

Breastfeeding is the ideal feeding modality for child growth and development^1^, being recommended as the only feeding source in the first six months of life and supplemented by healthy foods up to two years of life or more^a^. However, the threat of a multibillion-dollar industry that competes directly with breastfeeding^2^ and the excessive marketing of infant formulas and childcare-related products has led to thousands of babies dying of malnutrition and ingestion of infant formulas prepared with contaminated water^3^ and hinders mothers’ ability to choose the best way to feed their children^3^.

The World Health Organization (WHO) launched in 1981 the International Code of Marketing of Breast-milk Substitutes in response to this threat, aiming at restricting the marketing of these products^b^. Based on it and in line with the National Breastfeeding Promotion Program^4^, the Brazilian Health Council adopted in 1988 a code that prohibits advertising and commercial promotion of infant formulas, baby bottles and nipples^c^.

In the following decades, the Brazilian Ministry of Health and the Brazilian Health Regulatory Agency (ANVISA)^d , e^ expanded the scope of this code, which came to be called *Norma Brasileira de Comercialização de Alimentos para Lactentes e Crianças de Primeira Infância, Bicos, Chupetas e Mamadeiras* (NBCAL – Brazilian Code of Marketing of Infant and Toddlers Food and Childcare-related products). ANVISA, state and municipal regulatory agencies are responsible for monitoring companies and commercial establishments’ compliance with the guidelines and the adoption of actions applicable to infringers^5^. In 2006, NBCAL was strengthened as Law No. 11,265^f^, regulated in 2015 by Decree No. 8,552, and it was called Brazilian Code of Marketing of Infant and Toddlers Food and Childcare-related products^g^. This Law prohibits any form of commercial promotion of infant formulas, bottle nipples, pacifiers, baby bottles and nipple protectors^g^.

Despite NBCAL’s national scope and importance to public health, systematic efforts to monitor this standard by public authorities are scarce. The only record found was of 2006, when ANVISA promoted national monitoring involving higher education institutions and state health regulators, finding numerous violations of NBCAL^6^. Non-governmental organizations such as the International Baby-Food Action Network (IBFAN network) have voluntarily assumed the role of monitoring the code, with a methodology that seeks to detect new forms of violation^h^, but that does not reflect their extent.

Based on the lack of official data and knowledge of the prevalence of infringements, our study aimed at evaluating the compliance with NBCAL in the commercialization of infant formulas, bottle nipples, pacifiers, baby bottles and nipple protectors, for which commercial promotion is prohibited by law, in a pioneering and systematized way, using a census of commercial establishments in a geographical region of the city of Rio de Janeiro (RJ).

## METHOD

A cross-sectional study included in the survey “Evaluation of the compliance with the Brazilian Code of Marketing of Infant Formulas in commercial establishments and health services.” A census of all pharmacies, supermarkets and department stores that sold products covered by NBCAL in the South Zone of Rio de Janeiro was conducted through direct observation and interviews with managers of establishments. Butcheries, bakeries, exclusively manipulation and homeopathic pharmacies were excluded.

The city of Rio de Janeiro is located in the Southeast Region and is the second largest Brazilian metropolis, which has ten Health Planning Areas (AP). AP 2.1 was selected, corresponding to the South Zone, which has an extensive and diversified commercial network to serve a population distributed among 18 middle and high-income neighborhoods and favelas. A list of pharmacies, supermarkets and department stores by neighborhood and their addresses was obtained from TeleListas^i^ and complemented by search on websites of the main corresponding chains.

Data were collected by seven health professionals previously trained in NBCAL^4^. A pilot study was conducted in February 2017 in neighborhoods in the North Zone of Rio de Janeiro and the city of Niteroi to train interviewers, improve data collection instruments and define field strategy.

Data collection instrument was adapted from a form developed by the IBFAN^h^ network and complemented with questions related to the marketing of products and the profile of the interviewees. The adaptation of this form sought to enable data collection by electronic means. We used the Magpi application, which is a data collection and visualization platform designed for mobile applications^7^. The electronic form included the identification of the type of commercial establishment, the products marketed addressed in NBCAL, and the presence and qualification of the infringement. Establishments’ managers were interviewed about their familiarity with NBCAL and the products addressed in this law with the same instrument, as well as the visits of companies’ representatives.

The fieldwork, conducted in March and April 2017 under the supervision of the researchers responsible for the project, consisted in observing the establishments, marketed products and violations to NBCAL, and interviews with managers. We observed the commercial name of the product and the name of the manufacturer contained in the label to identify the products marketed with infringement.

The interviewers were previously assigned to collect data in different neighborhoods, thus avoiding the overlay of the collection. All establishments that marketed products addressed in NBCAL initially listed were evaluated by interviewers. This list was updated throughout fieldwork, as interviewers covered the streets of neighborhoods. Permanently closed establishments were excluded and establishments that were not on the list were included.

Our study analyzed the compliance with NBCAL regarding the marketing of all products addressed: infant formulas, follow-up formulas, baby bottles, bottle nipples, pacifiers and nipple protectors, of which commercial promotion is prohibited^4^. According to NBCAL, the price discounts or offers, special exposure on gondola tips or in highlighted displays and the distribution of promotional gifts or free samples of these items are prohibited^d , e , f , g^.

Data were exported and analyzed by the SPSS statistical program version 21 (Statistical Package for the Social Sciences). We created indicators to evaluate commercial practices, namely: 1. frequency of marketing by product groups and by type of commercial establishment; 2. prevalence of infringement by product group and by type of commercial establishment; 3. prevalence of each commercial promotion strategy by type of establishment; 4. prevalence of infractions of infant formulas and childcare-related products by company; 5. proportion of establishments’ managers that were familiar with NBCAL; 6. frequency of visits by commercial representatives of manufacturers of these products to establishments. We conducted descriptive analyses and their results were shown in tables.

Our study followed CNS resolutions no. 466/12 and no. 510/16^j , k^. NBCAL monitoring is a free and public practice, accessible to any citizen, and it does not require prior authorization from commercial establishments. The exemption of previous formal consent of commercial establishments was requested to the Research Ethics Committee (REC) of Universidade Federal Fluminense (UFF) since the application for such authorization could lead managers to change the environment by the withdrawal of illegal products and promotions to suit the law. We also requested the exemption of the signing of the informed consent form by the managers establishments because this procedure could expose them to sanctions by their companies. The interviewees were explained that the survey would not have a punitive character; confidentiality, anonymity, autonomy and freedom to refuse to participate was guaranteed, and free verbal consent was obtained. Our study was approved by the REC of UFF (opinion no. 1,878,013/2016) and supported by the *Conselho Nacional de Desenvolvimento Científico e Tecnológico* (CNPq) and the *Fundação de Amparo à Pesquisa do Estado do Rio de Janeiro* (Faperj).

## RESULTS

A total of 352 establishments in the South Zone of Rio de Janeiro were evaluated: 240 pharmacies, 88 supermarkets and 24 department stores. Almost 90% marketed products whose commercial promotion is prohibited by NBCAL; more than 80% marketed infant formulas and more than 70% childcare-related products. Several brands of infant formulas with denominations such as premium, supreme, comfor, profuture, proexpert, gentlease and advance produced by four companies, namely Nestlé, Danone, Mead Johnson e Abbot, were observed. Nearly every pharmacy marketed different infant formulas, bottle nipples, pacifiers, baby bottles and nipple protectors. More than half of supermarkets marketed infant formulas and less than 10% childcare-related products. Department stores did not sell infant formulas, only bottles, bottle nipples and pacifiers, as shown in Table 1 .

**Table 1:** Proportion of pharmacies, supermarkets and department stores that sold products whose commercial promotion is prohibited by NBCAL, according to the type of product. Zona Sul, Rio de Janeiro, 2017.

	Pharmacies		Supermarkets		Stores		Total	
			
	n	%	n	%	n	%	n	%
Infant Formula	231	96.2	51	56.0	0	0.0	282	80.2
Infant formula: first semester	227	94.6	51	58.8	0	0.0	278	79.0
Infant formula: second semester	225	93.8	45	51.1	0	0.0	270	76.7
Childcare-related products*	226	94.1	7	8.0	21	29.1	254	72.1
Bottle	221	92.1	5	5.7	21	87.5	247	70.2
Bottle nipple	212	88.3	3	3.4	18	75.0	233	66.2
Pacifier	219	91.3	4	4.5	18	75.0	241	68.5
Nipple protector	128	53.3	0	0.0	0	0.0	128	36.4
Establishments selling one or more of these products	237	98.8	53	60.3	21	87.5	311	88.3
Total establishments	240	100	88	100	24	100	352	100

NBCAL: Brazilian Code of Marketing of Infant and Toddlers Food and Childcare-related productsBrazilian Guidelines for Commercialization of Formulas for Infants and Children of Early Childhood and Childcare-related Products

* Childcare-related products: bottle nipples, baby bottles, pacifiers and nipple protectors.

Commercial promotions of infant formulas, bottle nipples, pacifiers, baby bottles and nipple protectors prohibited by NBCAL were verified in 63 establishments, which corresponds to 20.3% of the total that sold these products: 21.9% of pharmacies, 7.5% of supermarkets and 33.3% of department stores. Illegal commercial promotion of products with the highest prevalence of infractions of NBCAL were formulas for infants, found in 16.0% of the establishments. Illegal commercial promotions of bottle nipples, pacifiers, baby bottles and nipple protectors were found in 9.4% of the establishments. The most frequent commercial promotion strategies were discounts and special exposure, and one of the establishments offered promotional gifts, as shown in Table 2 .

**Table 2:** Proportion of pharmacies, supermarkets and department stores with illegal commercial promotion of infant formulas, bottle nipples, baby bottles, pacifiers and nipple protectors, and commercial promotion strategies, according to the type of establishment. Zona Sul, Rio de Janeiro, 2017.

	Pharmacies		Supermarkets		Stores		Total	
			
	n	%	n	%	n	%	n	%
Infant formulas								
Sold with illegal promotion	44	19.0	1	2.0	-	-	45	16.0
Total that sold	231		51		0		282	
Bottle nipples, baby bottles, pacifiers and nipple protectors								
Sold with illegal promotion	14	6.2	3	42.9	7	33.3	24	9.4
Total that sold	226		7		21		254	
Infant formulas, bottles, pacifiers, nipples and nipple protectors								
Sold with illegal promotion	52	21.9	4	7.5	7	33.3	63*	20.3
Sold without commercial promotion	185	78.1	49	92.5	14	66.7	248	79.7
Commercial promotion strategy practiced								
Discount	33	13.9	1	1.9	7	33.3	41	13.2
Special exposure	26	11.0	3	5.7	0	0	29	9.3
Promotional gift	1	0.4	0	0	0	0	1	0.3
Total that sold	237	100	53	100	21	100	311	100
They did not sell	3		35		3		41	

* 63 establishments illegally sold infant formulas and/or bottle nipples, baby bottles, pacifiers and nipple protectors.

Two thirds of the 45 establishments that marketed infant formulas irregularly had Nestlé products in this condition. This was the company with the highest frequency of illegally marketed infant formulas and infractions in a single establishment, reaching ten products with infractions. The company Danone also presented high rates, with up to six products with infringements in a single establishment. Almost half of the 24 establishments, in which bottle nipples, pacifiers, baby bottles and/or nipple protectors violations were observed, had Lillo products in this condition.. This was the company that had the most childcare-related products marketed with infractions and the highest frequency of products with infractions in a single establishment, as shown in Table 3 .

**Table 3:** Companies that produce infant formulas and childcare-related products with illegally marketed products among establishments with illegal commercial promotion. Zona Sul, Rio de Janeiro, 2017.

Companies	Establishments with infractions		Products with infractions	Number of infractions per establishment
	
	n	%	n	
Infant formulas				
Nestlé	30	66.7	110	up to 10
Danone	27	60.0	72	up to 6
Mead Johnson	18	40.0	30	up to 4
Abbott	5	11.1	6	up to 2
Others	1	2.2	1	up to 1
Total	45^b^		219	up to 18
Childcare products^a^				
Lillo	11	45.8	27	up to 6
Mam	6	25.0	13	up to 4
Nuk	6	25.0	10	up to 2
Kuka	4	16.7	16	up to 5
Neopan	2	8.3	6	up to 4
Others	6	25.0	7	up to 2
Total	24^c^		79	up to 9

^a^ bottle nipples, baby bottles, pacifiers and nipple protectors.

^b^ Total establishments with infant formulas on undue commercial promotion.

^c^ Total establishments with childcare-related products on undue commercial promotion.

Out of all establishments, 309 managers and pharmacists, were interviewed, 12.2% refused (n = 43), mainly among responsible for department stores (n = 12), who claimed they did not have authorization for interviews. More than half reported unfamiliarity with NBCAL, 55.7% in pharmacies, 35.5% in supermarkets and 58.7% in stores, while about a quarter reported having heard of it. When asked about NBCAL’s products, 46.1% cited infant formulas and less than 10% bottle nipples, pacifiers and baby bottles, as shown in Table 4 .

**Table 4:** Familiarity of the commercial manager with the NBCAL, according to the type of establishment. Zona Sul, Rio de Janeiro, 2017.

Familiarity level	Pharmacies		Supermarkets		Stores		Total	
			
	n	%	n	%	n	%	n	%
Unfamiliar	123	55.7	27	35.5	7	58.3	157	50.8
Heard about it	54	24.4	19	25.0	2	16.7	75	24.3
Familiar	44	19.9	30	39.5	3	25.0	77	24.9
They cited infant formulas	52	53.1	18	36.7	0	0	70^b^	46.1
Childcare-related products^a^	12	12.2	2	4.1	1	20.0	15^b^	9.9

NBCAL: Brazilian Code of Marketing of Infant and Toddlers Food and Childcare-related products

^a^ Childcare-related products: bottle nipples, baby bottles, pacifiers and nipple protectors. ^b^ Total managers of commercial establishments who cited infant formulas and childcare-related products, who reported familiarity with the law or, at least, having heard of the guidelines.

Three quarters of managers and pharmacists interviewed reported that Nestlé’s commercial representatives had visited the respective establishment daily, weekly, fortnightly or monthly, while the company Danone was cited by less than half of interviewees. Ten percent of respondents reported that Mam, a company that produces childcare-related products, sent commercial representatives to visit the establishment weekly, fortnightly or monthly, as shown in Table 5 .

**Table 5:** Proportion of pharmacies, supermarkets and department stores visited by companies of infant formulas and childcare-related products, according to those responsible for the establishments. Zona Sul, Rio de Janeiro, 2017.

Company	Pharmacies		Supermarkets		Stores		Total	
			
	n	%	n	%	n	%	n	%
Infant formulas								
Nestlé	162	67.5	67	76.1	5	2.1	234	75.7
Danone	103	43.0	37	42.0	0	0	140	45.3
Others	26	11.0	1	1.1	0	0	27	8.7
Childcare-related products*								
Mam	31	13.0	0	0	0	0	31	10.0
Lillo	18	7.5	1	1.1	1	4.2	20	6.5
Kuka	13	5.4	0	0	2	8.3	15	4.9
Nuk	13	5.4	0	0	0	0	13	4.2
Others	13	5.4	0	0	0	0	13	4.2
Total	240	100	88	100	24	100	352	100

* bottle nipples, baby bottles, pacifiers and nipple protectors

## DISCUSSION

More than a fifth of the commercial establishments in the South Zone of Rio de Janeiro were promoting infant formulas, bottle nipples and baby bottles, which has been considered illegal by Brazilian law for 30 years^c^. Promotions are considered strategies to induce retail sales such as special exposure, discount coupons, offers or reduced prices, premiums, promotional gifts, linked sales or special presentations^d , e , f , g^.

Pharmacies marketed all types of products whose commercial promotion is prohibited; most supermarkets marketed infant formulas and some marketed bottle nipples, baby bottles and pacifiers; department stores marketed only bottle nipples, baby bottles and pacifiers. Department stores and pharmacies were the commercial establishments with the highest percentage of infractions, followed by supermarkets. The most common promotional strategy was discount, followed by special exposure.

A study conducted in the municipality of Piracicaba (SP), in 2012, also observed that there were more discounts and special exposures of bottle nipples, baby bottles and pacifiers in pharmacies than in supermarkets^8^. Another study, conducted in the main supermarket chain of the municipality of Teresina (PI), in 2009, observed that half of the establishments presented infractions in the marketing of infant formulas, bottle nipples, baby bottles and pacifiers, especially through special exposure^9^. In a more recent study conducted in 25 supermarkets in the municipality of Mossoró (RN), in 2016, the commercial promotion of these products was observed in 12% of the supermarkets^10^, a percentage closest to that observed in our study. The variation in the proportion of infractions found in the studies may be due to the commercial pressure of industries and commercial practices to attract consumers in differentiated economic contexts, or due to the type of establishments included by convenience samples in the studies.

Considering the products evaluated, the infant formulas presented the highest frequency of illegal commercial promotion. The wide variety of brands of infant formulas refers to novelties and claims additional benefits to nutrition and baby health^11^ as a strategy for the expansion of the market for breast-milk substitute products^12^. Sales growth of these products exceeds 10% per year^13^ in low- and middle-income countries such as Brazil, corroborating infant morbidity and mortality from diarrhea, pneumonia and other infections^1^.

Nestlé and Danone were the manufacturers of infant formulas with the highest number of products marketed through illegal promotion and are also cited by the managers of commercial establishments as those that most frequently sent their representatives to carry out commercial promotion of their products. The consistency found between the number of products illegally marketed and the companies that visit the most cited establishments by managers did not occur among the childcare-related products, because Lillo was the company with the highest amount of illegally marketed products, while Mam was the most cited for sending commercial representatives to the establishments.

The large number of infractions found in establishments can be partially explained by managers’ unfamiliarity with the legislation. In a secondary data study conducted in 2000, 89.9% of pharmacy employees and 79.2% of supermarket employees claimed they were unaware of NBCAL^14^, while in our study, conducted more than fifteen years later, more than half of those responsible for pharmacies and department stores and more than a third of those responsible for supermarkets claimed the same, which indicates that the familiarity with the legislation is still insufficient.

Identifying the responsible for non-compliance with the law is extremely difficult, since companies seem to act through commercial establishments: industry representatives visit the establishments and seek to induce illegal promotion through different strategies. Although companies that make infant formulas, bottle nipples, baby bottles, pacifiers and nipple protectors became increasingly aware of NBCAL and adapted the labeling of their products as the law improved^15^, it does not seem to happen to the managers of the commercial establishments.

NBCAL is an important tool for legal protection of breastfeeding^5^ by regulating the marketing of infant formulas and products that interfere in breastfeeding^g^. Compliance and supervision of this legislation is officially attributed to municipal and state health regulatory agencies (Visa)^d , e , f^. However, there is only one record of a systematized monitoring action, promoted by Anvisa, which coordinated national monitoring^6^ in 2006. Monitoring actions of local “Visas” have been sporadic and ineffective in inhibiting the non-compliance of NBCAL by commercial establishments, a fact observed both by the high number of infractions detected in our study and by the lack of public records of monitoring^l^.

Burlandy et al.^16^report that private sector market practices seek both to stimulate the consumption of their products and to block government measures that affect their economic interests such as regulation and implementation of health-protection laws. On the one hand we have the marketing of manufacturers of infant formulas, bottle nipples, baby bottles and pacifiers, directed to the stimulus of consumption, and, on the other hand, an attempt to obstruct advances in laws that regulates its marketing, which can be observed by the more than nine-year gap between the enactment of Law 11,265^f^ and its regulation^g^. The lack of systematic monitoring of commercial establishments and educational actions on NBCAL by the competent bodies can be explained not only by the low coverage of specific monitoring of NBCAL by the regulatory agencies, but also by the lobby of industries. Therefore, conflicts of interest in the area of child feeding strongly impact the current scenario of non-compliance with NBCAL^l^.

We cite as study limitations the percentage of refusals of interviews by those responsible for commercial establishments, 12.2%, concentrated on chained department stores, which may have compromised findings related to managers’ familiarity of NBCAL and visits of companies’ representatives. The percentage of losses in the interview with those responsible for the establishments can change the census characteristic regarding the information they provided, while the evaluation of the commercial promotion conducted by observing pharmacies, supermarkets and department stores in neighborhoods and favelas was conducted in its entirety, without any loss.

Our study was very timely because it established a baseline of compliance with NBCAL in a geographic region, shortly after the law was regulated by Decree No. 8,552/15. Thus, the results of future studies can be compared over time, and the evolution of compliance with the guidelines can be verified, which is a law that protects mothers and infants against industry strategies.

Given the high prevalence of violations of NBCAL, and aiming at a greater compliance with Law no. 11,265^f^, intensifying educational actions is recommended to gain a greater familiarity with the law, as well as the effective monitoring by the responsible bodies and the application of the appropriate punishments established by law^m^. A further investigation into the causal chain of non-compliance with NBCAL is also recommended to enable a better understanding of the etiology of this phenomenon.

## Footnotes

a World Health Organization. Indicators for assessing infant and young child feeding practices. Geneva: WHO; 2010.

b World Health Organization. International code of marketing of breast-milk substitutes. Geneva: WHO; 1981.

c Ministério da Saúde (BR), Conselho Nacional de Saúde. Resolução Nº 5 de 20 de dezembro de 1988. Norma brasileira para comercialização de alimentos para lactentes. Diario Oficial Uniao. 23 dez 1988; Seção 1.

d Ministério da Saúde (BR), Agência Nacional de Vigilância Sanitária. Resoluçao RDC Nº 221, de 5 de agosto de 2002. Regulamento técnico sobre chupetas, bicos, mamadeiras e protetores de mamilo. Diario Oficial Uniao. 6 ago 2002; Seção 1:557-8.

e Ministério da Saúde (BR), Agência Nacional de Vigilância Sanitária. RDC nº 222 de 5 de agosto de 2002. Regulamento técnico para promoção comercial dos alimentos para lactentes e crianças de primeira infância. Diario Oficial Uniao. 6 ago 2002. Seção 1:558-60.

f Brasil. Lei n 11.265, de 3 de janeiro de 2006. Regulamenta a comercialização de alimentos para lactentes e crianças de primeira infância e também a de produtos de puericultura correlatos. Diario Oficial Uniao. 4 jan 2006; Seção 1:1-3.

g Brasil. Decreto Nº 8.552, de 3 de novembro de 2015. Regulamenta a Lei 11.265/2006, que dispõe sobre a comercialização de alimentos para lactentes e crianças de primeira infância e de produtos de puericultura correlatos. Diario Oficial Uniao. 4 nov 2015; Seção 1;5.

h Salve JM, De Divitis RMPF, Toma TS. Violando as Normas 2008: relatório nacional das violações à Norma Brasileira de Comercialização de Alimentos para Lactentes e Crianças de Primeira Infância, Bicos, Chupetas e Mamadeiras e Lei 11.265/2006: edição comemorativa dos 20 anos da NBCAL. Jundiaí, SP: IBFAN Brasil, 2008 [citado 11 out 2019]. Available from: http://www.ibfan.org.br/monitoramento/pdf/doc-360.pdf

i Available from: http://www.telelistas.net/rj/rio+de+janeiro .

j Ministério da Saúde (BR), Conselho Nacional de Saúde. Resolução Nº 510, de 7 de abril de 2016. Princípios éticos das pesquisas em ciências humanas e sociais. Brasília, DF; 2016 [citado 3 jan 2018]. Available from: http://bvsms.saude.gov.br/bvs/saudelegis/cns/2016/res0510_07_04_2016.html

k Ministério da Saúde (BR), Conselho Nacional de Saúde (Brasil). Resolução nº466, de 12 de dezembro de 2012. Aspectos éticos da pesquisa envolvendo seres humanos. Brasília, DF; 2012 [citado 3 jan 2018]. Available from: http://bvsms.saude.gov.br/bvs/saudelegis/cns/2013/res0466_12_12_2012.html

l Instituto Brasileiro de Defesa do Consumidor. Pesquisa Amamentação Desvalorizada. Rev IDEC. 2015;(195):22-4. Available from: https://www.idec.org.br/uploads/revistas_materias/pdfs/195-amamentacao1.pdf

m Brasil. Lei 6.437 de 20 de agosto de 1977. Configura infrações à legislação sanitária federal, estabelece sanções às respectivas, e dá outras providências. Brasília, DF;1977 [citado 3 jan 2018]. Available from: http://www.planalto.gov.br/ccivil_03/leis/l6437.htm
