# Supplementary material for: Illegal commercial promotion of products competing with breastfeeding
Source: Rev Saude Publica. 2020 Jan 23;54:10. doi: 10.11606/s1518-8787.2020054000854 (PMC6986861; doi:10.11606/s1518-8787.2020054000854)
Supplement: Translation

# Translation: Promoção comercial ilegal de produtos que competem com o aleitamento materno

**Authors:** Karine Borges da Silva, Maria Inês Couto de Oliveira, Cristiano Siqueira Boccolini, Enilce de Oliveira Fonseca Sally

**Affiliations:** I Brasil Universidade Federal Fluminense. Programa de Pós-graduação em Saúde Coletiva. Niterói, RJ, Brasil; II Brasil Universidade Federal Fluminense. Instituto de Saúde Coletiva. Departamento de Epidemiologia e Bioestatística. Niterói, RJ, Brasil; III Brasil Fundação Oswaldo Cruz. Instituto de Comunicação e Informação Científica e Tecnológica em Saúde. Laboratório de Informação em Saúde. Rio de Janeiro, RJ, Brasil; IV Brasil Universidade Federal Fluminense. Faculdade de Nutrição. Departamento de Nutrição Social. Niterói, RJ, Brasil

**Keywords:** Substitutos do Leite Humano, Fórmulas Infantis, Chupetas, Comercialização de Produtos, Publicidade Direta ao Consumidor, legislação & jurisprudência

## Abstract

**OBJETIVO:**

Avaliar se a comercialização de fórmulas infantis, mamadeiras, bicos, chupetas e protetores de mamilo é realizada em cumprimento com a Norma Brasileira de Comercialização de Alimentos para Lactentes e Crianças de Primeira Infância e de Produtos de Puericultura Correlatos (NBCAL). A promoção comercial desses produtos é proibida pela Lei 11.265.

**MÉTODOS:**

Estudo transversal conduzido em 2017 por meio de um censo de todas as farmácias, supermercados e lojas de departamento que comercializavam produtos abrangidos pela NBCAL na Zona Sul do Rio de Janeiro. Profissionais de saúde capacitados na NBCAL utilizaram formulário eletrônico estruturado para observação direta dos estabelecimentos e para entrevista com seus responsáveis. Foram criados seis indicadores de avaliação das práticas comerciais e realizadas análises descritivas.

**RESULTADOS:**

Foram avaliados 352 estabelecimentos comerciais: 240 farmácias, 88 supermercados e 24 lojas de departamento, dos quais 88% comercializavam produtos cuja promoção é proibida pela NBCAL. Foram encontradas promoções comerciais ilegais em 20,3% daqueles que comercializavam os produtos investigados: 52 farmácias (21,9%), quatro supermercados (7,5%) e sete lojas de departamento (33,3%). As estratégias de promoção comercial mais frequentes foram os descontos (13,2%) e as exposições especiais (9,3%). Os produtos com maior prevalência de infrações à NBCAL foram as fórmulas infantis (16,0%). Foram entrevistados 309 responsáveis por estabelecimentos comerciais, 50,8% relatando não conhecer a lei. Mais de três quartos dos responsáveis relataram receber visitas nos estabelecimentos de representantes comerciais de empresas fabricantes de fórmulas infantis.

**CONCLUSÃO:**

Mais de um quinto dos estabelecimentos comerciais faziam promoção comercial de fórmulas infantis para lactentes, mamadeiras e bicos, apesar de essa prática ser proibida no Brasil há trinta anos. É necessária a capacitação dos seus responsáveis. Os órgãos governamentais devem realizar fiscalização dos estabelecimentos comerciais para coibir estratégias de persuasão e indução à vendas desses produtos, garantindo às mães autonomia na decisão sobre a alimentação de seus filhos.

## INTRODUÇÃO

O aleitamento materno é a modalidade ideal de alimentação para o crescimento e desenvolvimento infantil^1^, sendo recomendado na forma exclusiva nos primeiros seis meses de vida e complementado por alimentos saudáveis até os dois anos de vida ou mais^a^. Contudo, a ameaça de uma indústria multibilionária que compete diretamente com o aleitamento materno^2^e o avanço do *marketing* abusivo de fórmulas infantis e produtos correlatos levou à morte milhares de bebês por desnutrição e ingestão de fórmulas infantis preparadas com água contaminada^3^e põe em risco a capacidade das mães de escolher a melhor forma de alimentar seus filhos^3^.

Em resposta a essa ameaça, a Organização Mundial da Saúde (OMS) lançou em 1981 o Código Internacional de Comercialização de Substitutos do Leite Materno, visando restringir o *marketing* desses produtos^b^. Com base nele e em consonância com o Programa Nacional de Incentivo ao Aleitamento Materno^4^, o Conselho Nacional de Saúde brasileiro adotou em 1988 uma norma que proíbe a propaganda e a promoção comercial de fórmulas infantis, mamadeiras e bicos^c^.

Nas décadas seguintes, o Ministério da Saúde e a Agência Nacional de Vigilância Sanitária (Anvisa)^d , e^ampliaram consecutivamente o escopo dessa norma, que passou a denominar-se Norma brasileira de comercialização de alimentos para lactentes e crianças de primeira infância, bicos, chupetas e mamadeiras (NBCAL), cabendo à Anvisa e às vigilâncias estaduais e municipais a fiscalização do seu cumprimento pelas empresas e estabelecimentos comerciais e a adoção das ações aplicáveis aos infratores^5^.

Em 2006, a NBCAL foi fortalecida enquanto Lei nº 11.265^f^, regulamentada em 2015 pelo Decreto nº 8.552, passando a ser denominada Norma Brasileira de Comercialização de Alimentos para Lactentes, Crianças de Primeira Infância e de Produtos de Puericultura Correlatos^g^. O conjunto dessa legislação proíbe qualquer forma de promoção comercial de fórmulas infantis para lactentes, bicos, chupetas, mamadeiras e protetores de mamilo^g^.

Contudo, apesar da abrangência nacional da NBCAL e da sua importância para a saúde pública, os esforços sistemáticos de monitoramento dessa norma pelas autoridades públicas são escassos. O único registro encontrado foi de 2006, quando a Anvisa promoveu um monitoramento nacional envolvendo instituições de ensino superior e vigilâncias sanitárias estaduais, encontrando inúmeras infrações à NBCAL^6^. Organizações não governamentais como a rede IBFAN ( *Internacional Baby-Food Action Network* ) têm assumido voluntariamente o papel de fiscalização da norma, com uma metodologia que busca detectar novas formas de violação^h^, mas que não reflete a extensão delas.

Em um cenário de ausência de dados oficiais e de desconhecimento da prevalência das infrações, o presente estudo teve como objetivo avaliar de forma pioneira e sistematizada o cumprimento da NBCAL na comercialização de fórmulas infantis, bicos, chupetas, mamadeiras e protetores de mamilo, para os quais a promoção comercial é proibida, utilizando um censo dos estabelecimentos comerciais de uma região geográfica da cidade do Rio de Janeiro (RJ).

## MÉTODO

Estudo transversal componente da pesquisa “Avaliação do cumprimento da Norma Brasileira de Comercialização de Alimentos para Lactentes em estabelecimentos comerciais e serviços de saúde”. Foi realizado censo de todas as farmácias, supermercados e lojas de departamento que comercializavam produtos abrangidos pela NBCAL na Zona Sul do Rio de Janeiro, por meio de observação direta e de entrevista com os responsáveis pelos estabelecimentos. Foram excluídos os açougues, as padarias, as farmácias exclusivamente de manipulação e as farmácias homeopáticas.

A cidade do Rio de Janeiro fica na Região Sudeste, é a segunda maior metrópole brasileira e possui dez Áreas de Planejamento em Saúde. Foi selecionada a AP 2.1, correspondente à Zona Sul, onde a rede comercial é extensa e diversificada para atender a uma população que se distribui por 18 bairros de renda média e alta e comunidades faveladas. Uma lista contendo a relação nominal das farmácias, supermercados e lojas de departamento por bairro, bem como seus endereços, foi obtida pela TeleListas^i^e complementada pela consulta aos sites das principais redes correspondentes.

A coleta de dados foi realizada por sete profissionais de saúde previamente capacitados em curso da NBCAL^4^. Foi realizado estudo-piloto, em fevereiro de 2017, em bairros da Zona Norte do Rio de Janeiro e da cidade de Niterói, para treinamento dos entrevistadores, aperfeiçoamento dos instrumentos de coleta de dados e definição da estratégia de campo.

O instrumento de coleta de dados foi adaptado de formulário desenvolvido pela rede IBFAN^h^, complementado com questões relativas à comercialização de produtos e ao perfil dos responsáveis entrevistados. A adaptação desse formulário visou possibilitar a coleta de dados por meio eletrônico, pelo aplicativo Magpi, uma plataforma de coleta e visualização de dados projetada para aplicativos móveis^7^. O formulário eletrônico contemplava a identificação do tipo de estabelecimento comercial, dos produtos comercializados de abrangência da NBCAL, e da presença e qualificação da infração, quando presente. Pelo mesmo instrumento, os responsáveis pelos estabelecimentos foram entrevistados quanto ao conhecimento da NBCAL e dos produtos abrangidos por essa lei e sobre as visitas de representantes das empresas fabricantes desses produtos.

O trabalho de campo, realizado nos meses de março e abril de 2017 sob supervisão dos pesquisadores responsáveis pelo projeto, constou da observação dos estabelecimentos, dos produtos comercializados e das infrações à NBCAL, além de entrevista com os responsáveis. Para identificar os produtos comercializados com infração, foram anotados o nome comercial do produto e o nome do fabricante, obtidos do rótulo.

Os entrevistadores foram designados previamente para coletar dados em bairros distintos uns dos outros, evitando a superposição da coleta. Todos os estabelecimentos que comercializavam produtos abrangidos pela NBCAL inicialmente listados foram avaliados pelos entrevistadores. Essa lista foi sendo atualizada à medida que os entrevistadores percorriam as ruas dos bairros durante o trabalho de campo. Estabelecimentos inexistentes no período da pesquisa foram excluídos e estabelecimentos que não constavam da lista foram acrescentados.

A pesquisa analisou o cumprimento da NBCAL quanto à comercialização de todos os produtos de sua abrangência: fórmulas infantis para lactentes, fórmulas infantis de seguimento para lactentes, mamadeiras, bicos, chupetas e protetores de mamilo, para os quais a promoção comercial é proibida^4^.

Segundo a NBCAL, é vedado o desconto de preços ou ofertas, exposição especial em pontas de gôndola ou em *displays* destacados e a distribuição de brindes ou amostras grátis desses itens^d , e , f , g^.

Os dados foram exportados e analisados pelo programa estatístico SPSS versão 21 ( *Statistical Package for the Social Sciences* ). Foram criados indicadores para avaliação das práticas comerciais, sendo eles: 1. frequência de comercialização de grupos de produtos por tipo de estabelecimento comercial; 2. prevalência de infração por grupo de produtos e por tipo de estabelecimento comercial; 3. prevalência de cada estratégia de promoção comercial por tipo de estabelecimento; 4. prevalência de infrações de fórmulas infantis e produtos de puericultura correlatos por fabricante; 5. proporção de responsáveis por estabelecimentos comerciais que conheciam a NBCAL; 6. frequência de visitas de representantes comerciais de empresas fabricantes desses produtos aos estabelecimentos. Foram conduzidas análises descritivas, e os resultados foram apresentados em forma de tabelas.

O presente estudo seguiu as resoluções CNS nº 466/12 e nº 510/16^j , k^. O monitoramento da NBCAL é uma prática livre e pública, acessível a qualquer cidadão, e não requer autorização prévia dos estabelecimentos comerciais. Foi solicitado ao Comitê de Ética em Pesquisa (CEP) da Universidade Federal Fluminense (UFF) a dispensa da anuência dos estabelecimentos comerciais, pois o requerimento de tal autorização poderia levar os responsáveis a alterar o ambiente da pesquisa, com retirada de produtos e de promoções ilegais para adequação à lei. Foi também solicitada a dispensa da assinatura de termo de consentimento livre e esclarecido pelos responsáveis pelos estabelecimentos, pois esse procedimento poderia expô-los a sanções por parte das empresas. Foi esclarecido aos entrevistados que a pesquisa não apresentaria caráter punitivo e foi garantido o sigilo, anonimato, autonomia e liberdade para recusa em participar, sendo obtido consentimento verbal livre. A pesquisa foi aprovada pelo CEP da UFF (parecer n^o^1.878.013/2016) e financiada pelo Conselho Nacional de Desenvolvimento Científico e Tecnológico (CNPq) e pela Fundação de Amparo à Pesquisa do Estado do Rio de Janeiro (Faperj).

## RESULTADOS

Foram avaliados 352 estabelecimentos da Zona Sul do Rio de Janeiro: 240 farmácias, 88 supermercados e 24 lojas de departamento. Quase 90% comercializavam produtos cuja promoção comercial é vedada pela NBCAL, mais de 80% comercializavam fórmulas infantis para lactentes e mais de 70% comercializavam produtos de puericultura correlatos. Diversas marcas de fórmulas infantis para lactentes foram observadas, rotuladas com denominações como *premium* , *supreme* , *comfor* , *profutura* , *proexpert* , *gentlease* e *advance* , produzidas por quatro empresas: Nestlé, Danone, Mead Johnson e Abbot. As farmácias, na sua quase totalidade, comercializavam variadas fórmulas infantis, bicos, chupetas, mamadeiras e protetores de mamilo. Mais da metade dos supermercados comercializava fórmulas infantis e menos de 10% comercializavam produtos de puericultura correlatos. As lojas de departamento não comercializavam fórmulas infantis, apenas mamadeiras, bicos e chupetas ( Tabela 1 ).

**Tabela 1:** Proporção de farmácias, supermercados e lojas de departamento que comercializavam produtos cuja promoção comercial é vedada pela NBCAL, segundo o tipo de produto. Zona Sul, Rio de Janeiro, 2017.

	Farmácias		Supermercados		Lojas		Total	
			
	n	%	n	%	n	%	n	%
Fórmula infantil para lactentes	231	96,2	51	56,0	0	0,0	282	80,2
Fórmula infantil: primeiro semestre	227	94,6	51	58,8	0	0,0	278	79,0
Fórmula infantil: segundo semestre	225	93,8	45	51,1	0	0,0	270	76,7
Produtos de puericultura correlatos*	226	94,1	7	8,0	21	29,1	254	72,1
Mamadeira	221	92,1	5	5,7	21	87,5	247	70,2
Bico	212	88,3	3	3,4	18	75,0	233	66,2
Chupeta	219	91,3	4	4,5	18	75,0	241	68,5
Protetor de mamilo	128	53,3	0	0,0	0	0,0	128	36,4
Estabelecimentos que comercializavam um ou mais desses produtos	237	98,8	53	60,3	21	87,5	311	88,3
Total de estabelecimentos	240	100	88	100	24	100	352	100

NBCAL: Norma Brasileira de Comercialização de Alimentos para Lactentes e Crianças de Primeira Infância e de Produtos de Puericultura Correlatos

* Produtos de puericultura correlatos: mamadeiras, bicos, chupetas e protetores de mamilo.

Promoções comerciais de fórmulas infantis para lactentes, fórmulas infantis de seguimento para lactentes, bicos, chupetas, mamadeiras e protetores de mamilo, proibidas pela NBCAL, foram verificadas em 63 estabelecimentos, o que corresponde a 20,3% do total que comercializava esses produtos: 21,9% das farmácias, 7,5% dos supermercados e 33,3% das lojas de departamento. Os produtos de promoção comercial ilegal com a maior prevalência de infrações à NBCAL foram as fórmulas infantis, encontradas em 16,0% dos estabelecimentos. Promoções comerciais ilegais de mamadeiras, chupetas, bicos e protetores de mamilo foram encontradas em 9,4% dos estabelecimentos. As estratégias de promoção comercial mais frequentes foram os descontos e as exposições especiais, e um dos estabelecimentos oferecia brinde ( Tabela 2 ).

**Tabela 2:** Proporção de farmácias, supermercados e lojas de departamento com promoção comercial ilegal de fórmulas infantis, mamadeiras, bicos, chupetas e protetores de mamilo e estratégias de promoção comercial, segundo o tipo de estabelecimento. Zona Sul, Rio de Janeiro, 2017.

	Farmácias		Supermercados		Lojas		Total	
			
	n	%	n	%	n	%	n	%
Fórmulas infantis para lactentes								
Comercializavam com promoção ilegal	44	19,0	1	2,0	-	-	45	16,0
Total que comercializava	231		51		0		282	
Mamadeiras, bicos, chupetas e protetores de mamilo								
Comercializavam com promoção ilegal	14	6,2	3	42,9	7	33,3	24	9,4
Total que comercializava	226		7		21		254	
Fórmulas infantis para lactentes, mamadeiras, chupetas, bicos e protetores de mamilo								
Comercializavam com promoção ilegal	52	21,9	4	7,5	7	33,3	63*	20,3
Comercializavam sem promoção comercial	185	78,1	49	92,5	14	66,7	248	79,7
Estratégia de promoção comercial praticada								
Desconto	33	13,9	1	1,9	7	33,3	41	13,2
Exposição especial	26	11,0	3	5,7	0	0	29	9,3
Brinde	1	0,4	0	0	0	0	1	0,3
Total que comercializava	237	100	53	100	21	100	311	100
Não comercializavam	3		35		3		41	

* 63 estabelecimentos comercializavam com promoção ilegal fórmulas infantis para lactentes e/ou mamadeiras, bicos, chupetas e protetores de mamilo.

Dois terços dos 45 estabelecimentos que comercializavam fórmulas infantis de maneira irregular tinham produtos da empresa Nestlé nessa condição. Essa foi a empresa com a maior frequência de fórmulas infantis comercializadas de forma ilegal e de infrações em um mesmo estabelecimento, chegando a dez produtos com infração em um único estabelecimento. A empresa Danone também apresentou índices elevados, tendo até seis produtos com infrações em um único estabelecimento. Quase a metade dos 24 estabelecimentos em que foram observadas infrações de bicos, chupetas, mamadeiras e/ou protetores de mamilo tinham produtos da empresa Lillo nessa condição. Essa foi a empresa que teve mais produtos de puericultura correlatos comercializados com infrações e a maior frequência de produtos com infrações em um mesmo estabelecimento ( Tabela 3 ).

**Tabela 3:** Empresas fabricantes de fórmulas infantis e de produtos de puericultura correlatos com produtos comercializados ilegalmente entre os estabelecimentos com promoção comercial indevida. Zona Sul, Rio de Janeiro, 2017.

Empresas	Estabelecimentos com infrações		Produtos com infrações	Nº de infrações por estabelecimento
	
	n	%	n	
Fórmulas infantis				
Nestlé	30	66,7	110	até 10
Danone	27	60,0	72	até 6
Mead Johnson	18	40,0	30	até 4
Abbott	5	11,1	6	até 2
Outros	1	2,2	1	até 1

Total	45^b^		219	até 18

Produtos de puericultura^a^				
Lillo	11	45,8	27	até 6
Mam	6	25,0	13	até 4
Nuk	6	25,0	10	até 2
Kuka	4	16,7	16	até 5
Neopan	2	8,3	6	até 4
Outros	6	25,0	7	até 2

Total	24^c^		79	até 9

^a^mamadeiras, bicos, chupetas e protetores de mamilo.

^b^Total de estabelecimentos com fórmulas infantis em promoção comercial indevida.

^c^Total de estabelecimentos com produtos de puericultura em promoção comercial indevida.

Foram entrevistados 309 responsáveis por estabelecimentos comerciais, entre gerentes e farmacêuticos, ocorrendo 12,2% de recusas (n = 43), principalmente entre responsáveis por lojas de departamento (n = 12), que alegaram não dispor de autorização da matriz para conceder entrevista. Mais da metade dos responsáveis entrevistados relataram não conhecer a NBCAL, 55,7% em farmácias, 35,5% em supermercados e 58,7% em lojas, enquanto cerca de um quarto já tinha ouvido falar dela. Quando perguntados sobre os produtos de abrangência da NBCAL, 46,1% citaram as fórmulas infantis e menos de 10% as mamadeiras, chupetas e bicos ( Tabela 4 ).

**Tabela 4:** Conhecimento do responsável comercial sobre a NBCAL, segundo o tipo de estabelecimento. Zona Sul, Rio de Janeiro, 2017.

Grau de conhecimento	Farmácias		Supermercados		Lojas		Total	
			
	n	%	n	%	n	%	n	%
Não conhece	123	55,7	27	35,5	7	58,3	157	50,8
Já ouviu falar	54	24,4	19	25,0	2	16,7	75	24,3
Sim	44	19,9	30	39,5	3	25,0	77	24,9
Citaram fórmulas infantis	52	53,1	18	36,7	0	0	70^b^	46,1
Citaram produtos de puericultura^a^	12	12,2	2	4,1	1	20,0	15^b^	9,9

NBCAL: Norma Brasileira de Comercialização de Alimentos para Lactentes e Crianças de Primeira Infância e de Produtos de Puericultura Correlatos

^a^Produtos de puericultura: mamadeiras, bicos, chupetas e protetores de mamilo.^b^Total de responsáveis pelos estabelecimentos comerciais que citaram fórmulas infantis e produtos de puericultura entre os que relataram conhecer ou já ter ouvido falar da Norma.

Três quartos dos responsáveis entrevistados relataram que representantes comerciais da empresa Nestlé visitavam o respectivo estabelecimento, com visitas diárias, semanais, quinzenais ou mensais, enquanto a empresa Danone foi citada por menos da metade dos responsáveis. Dez por cento dos entrevistados relataram que a Mam, empresa fabricante de produtos de puericultura correlatos enviava representantes comerciais para visitar o estabelecimento semanalmente, quinzenalmente ou mensalmente ( Tabela 5 ).

**Tabela 5:** Proporção de farmácias, supermercados e lojas de departamento visitados por empresas de fórmulas infantis e de produtos de puericultura correlatos, segundo os responsáveis pelos estabelecimentos. Zona Sul, Rio de Janeiro, 2017.

Empresa	Farmácias		Supermercados		Lojas		Total	
			
	n	%	n	%	n	%	n	%
Fórmulas infantis								
Nestlé	162	67,5	67	76,1	5	2,1	234	75,7
Danone	103	43,0	37	42,0	0	0	140	45,3
Outras	26	11,0	1	1,1	0	0	27	8,7
Produtos de puericultura*								
Mam	31	13,0	0	0	0	0	31	10,0
Lillo	18	7,5	1	1,1	1	4,2	20	6,5
Kuka	13	5,4	0	0	2	8,3	15	4,9
Nuk	13	5,4	0	0	0	0	13	4,2
Outras	13	5,4	0	0	0	0	13	4,2
Total	240	100	88	100	24	100	352	100

* mamadeiras, bicos, chupetas e protetores de mamilo.

## DISCUSSÃO

Mais de um quinto dos estabelecimentos comerciais da Zona Sul do Rio de Janeiro fazia promoção de fórmulas infantis para lactentes, além de mamadeiras e bicos, o que é considerado ilegal pela legislação brasileira há 30 anos^c^. São consideradas promoções as estratégias para induzir vendas no varejo, tais como exposições especiais, cupons de descontos, ofertas ou preços reduzidos, prêmios, brindes, vendas vinculadas ou apresentações especiais^d , e , f , g^.

Farmácias comercializavam todos os tipos de produtos cuja promoção comercial é proibida; a maioria dos supermercados comercializava fórmulas infantis e alguns vendiam mamadeiras, bicos e chupetas; já as lojas de departamento comercializavam apenas mamadeiras, bicos e chupetas. As lojas de departamento e as farmácias foram os estabelecimentos comerciais com o maior percentual de infrações, seguidas dos supermercados, e a estratégia promocional mais comum foi o desconto de preço, seguido da exposição especial.

Um estudo realizado em Piracicaba (SP) em 2012 também observou que nas farmácias havia mais descontos e exposições especiais de mamadeiras, bicos e chupetas do que nos supermercados^8^. Outro estudo, realizado na principal rede de supermercados de Teresina (PI) no ano de 2009, observou que metade dos estabelecimentos apresentava infrações na comercialização de fórmulas infantis para lactentes, mamadeiras, bicos e chupetas, em especial por meio de exposições especiais^9^. Em estudo mais recente, conduzido em 25 supermercados na cidade de Mossoró (RN), em 2016, foi observada promoção comercial desses produtos em 12% dos supermercados^10^, percentual mais próximo ao verificado no presente estudo. A variação na proporção de infrações encontradas nos estudos pode ser decorrente da pressão comercial das indústrias e das práticas comerciais para atrair consumidores presentes em contextos econômicos diferenciados, ou ainda da composição dos estabelecimentos incluídos nos estudos, por amostras de conveniência.

Considerando os produtos avaliados, as fórmulas infantis apresentaram a maior frequência de promoção comercial ilegal. A grande variedade de marcas de fórmulas infantis encontrada, que remetem a novidades e alegam benefícios adicionais à nutrição e saúde do bebê^11^, são uma estratégia para a expansão do mercado de produtos substitutos do leite materno^12^. Em países de baixa e média renda, como o Brasil, o crescimento das vendas desses produtos ultrapassa 10% ao ano^13^, corroborando para a morbimortalidade infantil por diarreia, pneumonia e outras infecções^1^.

Nestlé e Danone foram as fabricantes de fórmulas infantis com maior quantidade de produtos vendidos por meio de promoção ilegal, sendo também citadas pelos responsáveis pelos estabelecimentos comerciais como as que mais frequentemente enviavam seus representantes comerciais para realizar promoção comercial de seus produtos. A consistência encontrada entre a quantidade de produtos comercializados de forma ilegal e as empresas que visitam os estabelecimentos mais citadas pelos gerentes não ocorreu entre os produtos de puericultura correlatos, pois a empresa com maior quantidade de produtos comercializados ilegalmente foi a Lillo, enquanto a Mam foi a mais citada por enviar representantes comerciais aos estabelecimentos.

O grande número de infrações encontradas nos estabelecimentos pode ser explicado parcialmente pela falta de conhecimento da legislação por parte dos seus responsáveis. Em estudo de dados secundários realizado no ano de 2000, 89,9% dos funcionários de farmácias e 79,2% dos funcionários de supermercados alegaram desconhecer a NBCAL^14^, enquanto no presente estudo, realizado mais de quinze anos depois, mais da metade dos responsáveis por farmácias e por lojas de departamento e mais de um terço dos responsáveis por supermercados afirmaram o mesmo, indicando que o conhecimento da legislação ainda é insuficiente.

É extremamente complexo identificar a responsabilidade pelo descumprimento da lei, pois as empresas parecem agir por meio dos estabelecimentos comerciais: os representantes das indústrias visitam os estabelecimentos e buscam induzir a promoção ilegal por diversas estratégias. Apesar de as empresas fabricantes de fórmulas infantis, mamadeiras, bicos, chupetas e protetores de mamilo terem tomado conhecimento da NBCAL para adequação da rotulagem dos seus produtos à medida que a legislação vinha sendo aperfeiçoada^15^, o mesmo não parece acontecer com os gerentes dos estabelecimentos comerciais.

A NBCAL é um importante instrumento de proteção legal do aleitamento materno^5 , 6^por regulamentar a comercialização de alimentos e produtos infantis que interferem na prática da amamentação^g^. O cumprimento e fiscalização dessa legislação é atribuição oficial das agências de vigilância sanitária (Visa) municipais e estaduais^d , e , f^. Contudo, tem-se apenas um registro de ação sistematizada de fiscalização da NBCAL, promovida pela Anvisa, que coordenou um monitoramento nacional^6^em 2006. As ações de fiscalização das Visa locais têm sido esporádicas e pouco efetivas em inibir o descumprimento da NBCAL pelos estabelecimentos comerciais, fato constatado tanto pelo elevado número de infrações observado nessa pesquisa quanto pela falta de registros públicos desse controle^l^.

Burlandy et al.^16^relatam que as práticas mercadológicas do setor privado visam tanto o estímulo ao consumo dos seus produtos quanto o bloqueio a medidas governamentais que afetam seus interesses econômicos, como a regulamentação e implementação de legislações de proteção à saúde. Assim, temos por um lado o *marketing* das empresas fabricantes de fórmulas infantis, mamadeiras, bicos e chupetas, direcionado ao estímulo do consumo, e, por outro lado, uma tentativa de obstrução dos avanços na legislação que regula sua comercialização, que pode ser observada pela lacuna de mais de nove anos entre a promulgação da Lei 11.265^f^e a sua regulamentação^g^. A ausência de fiscalização sistemática dos estabelecimentos comerciais e de ações educativas sobre a NBCAL pelos órgãos competentes pode ser explicada não só pela baixa cobertura de vigilância específica da NBCAL por esses órgãos, como também pelo *lobby* das indústrias. Assim, os conflitos de interesses na área de alimentação infantil impactam fortemente o atual cenário de descumprimento da NBCAL^l^.

Quanto às limitações do presente estudo, cabe referir que o percentual de recusas de entrevistas pelos responsáveis por estabelecimentos comerciais foi de 12,2% e se concentrou principalmente nas lojas de departamento pertencentes a grandes redes, o que pode comprometer a generalização dos achados relativos ao conhecimento dos gerentes sobre a NBCAL e às práticas de visitas comerciais das empresas para esse setor específico do comércio. Esse percentual de perdas na entrevista com os responsáveis pelos estabelecimentos pode desconfigurar a característica de censo no tocante às informações fornecidas por eles, enquanto a avaliação da promoção comercial realizada mediante observação das farmácias, supermercados e lojas de departamento nos bairros e nas favelas foi realizada na sua totalidade, sem nenhuma perda.

O presente estudo foi bastante oportuno por ter estabelecido uma linha de base do cumprimento da NBCAL em uma região geográfica, pouco tempo após a lei ter sido regulamentada pelo Decreto Nº. 8.552/15. Com isso, os resultados de futuros estudos terão a possibilidade de comparação no decorrer do tempo, podendo ser verificada a evolução do cumprimento da norma, legislação que protege mães e lactentes contra as estratégias da indústria direcionadas a esse público tão vulnerável.

Diante da alta prevalência de infrações à NBCAL observada, e com vistas a um maior cumprimento da Lei nº 11.265^f^pelos estabelecimentos comerciais, recomenda-se a intensificação das ações educacionais para ampliar o conhecimento dessa legislação tão duramente conquistada pela sociedade civil, bem como a fiscalização efetiva de seu cumprimento pelos órgãos responsáveis e a aplicação das punições cabíveis em lei aos estabelecimentos que a infringirem^m^. Recomenda-se também uma maior investigação sobre a cadeia de causalidade do descumprimento da NBCAL, para que a etiologia desse fenômeno seja mais bem conhecida.

## Footnotes

a World Health Organization. Indicators for assessing infant and young child feeding practices. Geneva: WHO; 2010.

b World Health Organization. International code of marketing of breast-milk substitutes. Geneva: WHO; 1981.

c Ministério da Saúde (BR), Conselho Nacional de Saúde. Resolução Nº 5 de 20 de dezembro de 1988. Norma brasileira para comercialização de alimentos para lactentes. Diario Oficial Uniao. 23 dez 1988; Seção 1.

d Ministério da Saúde (BR), Agência Nacional de Vigilância Sanitária. Resoluçao RDC Nº 221, de 5 de agosto de 2002. Regulamento técnico sobre chupetas, bicos, mamadeiras e protetores de mamilo. Diario Oficial Uniao. 6 ago 2002; Seção 1:557-8.

e Ministério da Saúde (BR), Agência Nacional de Vigilância Sanitária. RDC nº 222 de 5 de agosto de 2002. Regulamento técnico para promoção comercial dos alimentos para lactentes e crianças de primeira infância. Diario Oficial Uniao. 6 ago 2002. Seção 1:558-60.

f Brasil. Lei n 11.265, de 3 de janeiro de 2006. Regulamenta a comercialização de alimentos para lactentes e crianças de primeira infância e também a de produtos de puericultura correlatos. Diario Oficial Uniao. 4 jan 2006; Seção 1:1-3.

g Brasil. Decreto Nº 8.552, de 3 de novembro de 2015. Regulamenta a Lei 11.265/2006, que dispõe sobre a comercialização de alimentos para lactentes e crianças de primeira infância e de produtos de puericultura correlatos. Diario Oficial Uniao. 4 nov 2015; Seção 1;5.

h Salve JM, De Divitis RMPF, Toma TS. Violando as Normas 2008: relatório nacional das violações à Norma Brasileira de Comercialização de Alimentos para Lactentes e Crianças de Primeira Infância, Bicos, Chupetas e Mamadeiras e Lei 11.265/2006: edição comemorativa dos 20 anos da NBCAL. Jundiaí, SP: IBFAN Brasil, 2008 [citado 11 out 2019]. Disponível em: http://www.ibfan.org.br/monitoramento/pdf/doc-360.pdf

i Disponível em http://www.telelistas.net/rj/rio+de+janeiro .

j Ministério da Saúde (BR), Conselho Nacional de Saúde. Resolução Nº 510, de 7 de abril de 2016. Princípios éticos das pesquisas em ciências humanas e sociais. Brasília, DF; 2016 [citado 3 jan 2018]. Disponível em: http://bvsms.saude.gov.br/bvs/saudelegis/cns/2016/res0510_07_04_2016.html

k Ministério da Saúde (BR), Conselho Nacional de Saúde (Brasil). Resolução nº466, de 12 de dezembro de 2012. Aspectos éticos da pesquisa envolvendo seres humanos. Brasília, DF; 2012 [citado 3 jan 2018]. Disponível em: http://bvsms.saude.gov.br/bvs/saudelegis/cns/2013/res0466_12_12_2012.html

l Instituto Brasileiro de Defesa do Consumidor. Pesquisa Amamentação Desvalorizada. Rev IDEC. 2015;(195):22-4. Disponível em: https://www.idec.org.br/uploads/revistas_materias/pdfs/195-amamentacao1.pdf

m Brasil. Lei 6.437 de 20 de agosto de 1977. Configura infrações à legislação sanitária federal, estabelece sanções às respectivas, e dá outras providências. Brasília, DF;1977 [citado 3 jan 2018]. Disponível em: http://www.planalto.gov.br/ccivil_03/leis/l6437.htm
